# Complete Genome Sequence Analysis of an Imported Dengue Virus Serotype 1 Strain from Myanmar

**DOI:** 10.1128/genomeA.00585-18

**Published:** 2018-07-05

**Authors:** Zhaoping Zeng, Jiandong Shi, Xiaofang Guo, Ling Mo, Ningzhu Hu, Hongning Zhou, Yunzhang Hu

**Affiliations:** a Institute of Medical Biology, Chinese Academy of Medical Sciences and Peking Union Medical College, Kunming, China; b Yunnan Key Laboratory of Vaccine Research and Development of Severe Infectious Disease, Kunming, China; c Yunnan Provincial Center of Arborvirus Research, Yunnan Provincial Key Laboratory of Vector-Borne Disease Control and Research, Yunnan Institute of Parasitic Diseases, Pu’er, China

## Abstract

It has been determined that recent dengue virus epidemics in Yunnan, China, originated from Southeast Asian strains. Here, we report the first complete genome sequence and molecular characterization of the imported dengue virus serotype 1 strain YNPE1. Phylogenetic analysis revealed that strain YNPE1 belonged to genotype I.

## Abstract (precis)

It has been determined that recent dengue virus epidemics in Yunnan, China, originated from Southeast Asian strains. Here, we report the first complete genome sequence and molecular characterization of the imported dengue virus serotype 1 strain YNPE1.

## GENOME ANNOUNCEMENT

Dengue virus (DENV) belongs to the genus Flavivirus in the family Flaviviridae (1). It is an endemic arbovirus affecting more than 390 million people worldwide (2). DENV is transmitted by female Aedes aegypti and A. albopictus mosquitos in tropical and subtropical regions (3). DENV has a single-stranded, positive-sense RNA genome that is approximately 11 kb in length, which includes a single open reading frame (ORF) encoding three structural proteins and seven nonstructural proteins (4). The ORF is flanked by the 5' nontranslated region (NTR), which is capped with a type I 7-methyl guanosine structure, and by the 3' NTR, with no polyadenylation (5). There are four antigenically distinct serotypes of DENV (DENV-1 to DENV-4), which are further clustered into distinct genotypes due to their high genetic mutation rates (6). Infection with any serotype of DENV can cause serious viral diseases in humans, including dengue fever (DF), dengue hemorrhagic fever (DHF), and dengue shock syndrome (DSS) (7).

As has been reported, recent DENV epidemics in mainland China were caused by imported strains (8). The threat of further DENV epidemics in China is aggravated by increased international travel, as well as environmental and climate factors (9). Yunnan Province lies in southwestern China and includes tropical and subtropical regions and borders three countries where DENV is hyperendemic, namely, Vietnam, Laos, and Myanmar. Sporadic epidemics caused by imported strains from these neighboring countries have been reported frequently over the past few decades (10). However, since 2013, large-scale DENV outbreaks have taken place in Dehong and Xishuangbanna prefectures of Yunnan Province (11). It is worth noting that most of the previous research on the molecular evolution of the imported DENV-1 strains was based on partial sequencing of the envelope gene (11, 12). Full-length genome information of DENV-1 strains imported from neighboring countries, especially Myanmar, which would be useful for viral surveillance, is still scarce.

Here, we report the first full-length genome sequence of an imported DENV-1 strain, named YNPE1, which was isolated from the serum of a 45-year-old female dengue patient returning from Yangon, Myanmar, on 1 September 2015. The viral genome was sequenced according to Illumina’s standard sequencing protocol. The complete genome sequence of strain YNPE1 is 10,821 nucleotides (nt) in length. The ORF encodes a polyprotein of 3,392 amino acids and is flanked by a 150-nt 5' NTR and a 492-nt 3' NTR. Phylogenetic analysis revealed that strain YNPE1 belonged to genotype I and clustered with the Chinese strain DENV-1/China/YN/15DGR401(2015) (GenBank accession number KX056473) imported from Myanmar (12), two strains isolated from Myanmar (GenBank accession numbers KX357969 and KX357938), the Sri Lankan strain DK87 (GenBank accession number KP398852), and the Thai strain DENV-1/TH/BID-V2270/2001 (GenBank accession number FJ687427). The results implied that the newly isolated DENV-1 strain was indeed imported from Myanmar and closely related to the Sri Lankan strain DK87 and the Thai strain TH/BID-V2270.

Overall, the full-length genome sequence and molecular evolutionary characteristics of the newly isolated DENV-1 strain YNPE1 described here will prompt surveillance and control of dengue epidemics in the Yunnan Province of China.

### Accession number(s).

The complete genome sequence of strain YNPE1 was deposited in GenBank under the accession number MF405201.
